# Water-Related Infrastructure in a Region of Post-Earthquake Haiti: High Levels of Fecal Contamination and Need for Ongoing Monitoring

**DOI:** 10.4269/ajtmh.14-0165

**Published:** 2014-10-01

**Authors:** Jocelyn M. Widmer, Thomas A. Weppelmann, Meer T. Alam, B. David Morrissey, Edsel Redden, Mohammed H. Rashid, Ulrica Diamond, Afsar Ali, Madsen Beau De Rochars, Jason K. Blackburn, Judith A. Johnson, J. Glenn Morris

**Affiliations:** Urban Affairs and Planning, School of Public and International Affairs, Virginia Tech University, Blacksburg, Virginia; Emerging Pathogens Institute, University of Florida, Gainesville, Florida; Department of Environmental and Global Health, College of Public Health and Health Professions, University of Florida, Gainesville, Florida; Christianville Foundation, Palatka, Florida; Spatial Epidemiology and Ecology Research Laboratory, Department of Geography, College of Liberal Arts and Sciences, University of Florida, Gainesville, Florida; Department of Health Services Research, Management and Policy, College of Public Health and Health Professions, University of Florida, Gainesville, Florida; Department of Pathology, Immunology, and Laboratory Medicine, College of Medicine, University of Florida, Gainesville, Florida; Department of Medicine, College of Medicine, University of Florida, Gainesville, Florida

## Abstract

We inventoried non-surface water sources in the Leogane and Gressier region of Haiti (approximately 270 km^2^) in 2012 and 2013 and screened water from 345 sites for fecal coliforms and *Vibrio cholerae*. An international organization/non-governmental organization responsible for construction could be identified for only 56% of water points evaluated. Sixteen percent of water points were non-functional at any given time; 37% had evidence of fecal contamination, with spatial clustering of contaminated sites. Among improved water sources (76% of sites), 24.6% had fecal coliforms versus 80.9% in unimproved sources. Fecal contamination levels increased significantly from 36% to 51% immediately after the passage of Tropical Storm Sandy in October of 2012, with a return to 34% contamination in March of 2013. Long-term sustainability of potable water delivery at a regional scale requires ongoing assessment of water quality, functionality, and development of community-based management schemes supported by a national plan for the management of potable water.

## Introduction

The January 12, 2010 earthquake that struck Haiti damaged an estimated 80–90% of the infrastructure in the city of Leogane^1^ and some 40–50% of the buildings in the neighboring town of Gressier.^2^ Provision of safe water has been a particular focus of the international organizations (IOs) and non-governmental organizations (NGOs) investing in the reconstruction efforts of this area. The perceived need for access to water in the months after the earthquake was grounded in the fact that Haiti has been reported to have the lowest coverage of improved water points in the region, with 73% of the urban population with access to an improved source and 47% of the rural population with access to an improved source.^3^ Even greater emphasis was placed on the need for safe, potable water with the introduction of cholera into Haiti in October of 2010.

**Figure 1.**
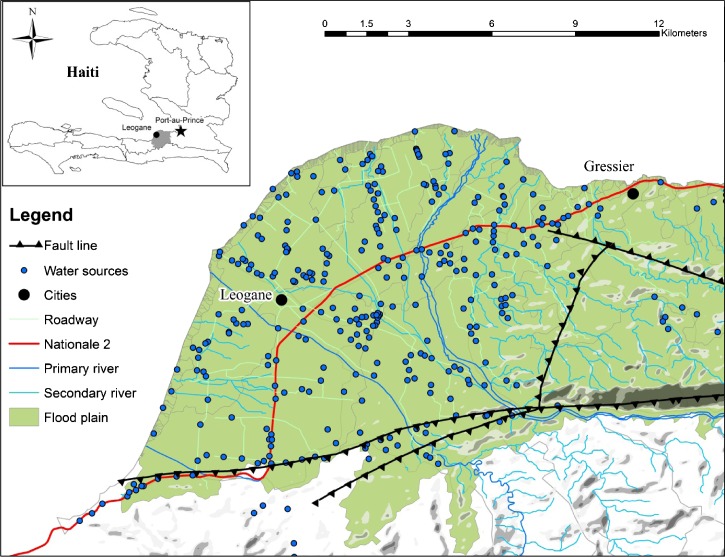
Map of study region. Topology of study region with water sources shown by blue dots.

In Haiti, including the cities of Leogane and Gressier, the result was the installation of hundreds of water points (Figure 1) by what is estimated to be over 23 IOs and NGOs. Rapid and poorly documented installation prompted a number of efforts to inventory these water points within 1 year of the 2010 earthquake, with efforts continuing to the present. Inventory efforts range from intraorganizational, where one national organization/IO sought to map the geographic localities of their own newly installed water points, to interorganizational, where groups of individuals have attempted to amass this information from multiple organizations into an open-source format, to governmental, with the recent efforts of the National Directorate for Water Supply and Sanitation (DINEPA) to create community-based water point inventory teams in the 10 departments of Haiti.

This study builds on a significant interorganizational web-based collaborative mapping effort that occurred between June and December of 2011 (the Collaborative Cholera Mapping Project [CCMP]) in the region surrounding Leogane and Gressier. This area of approximately 270 km^2^ is spatially heterogeneous, with high population densities centered within two urban nodes and sparsely populated outlying coastal and mountainous areas (Figure 1). Extrapolating from recent pre-earthquake census data, we estimate the population of the study area to be approximately 250,000.^4^ Route Nationale 2 runs through the region as the main artery feeding the urban centers of Leogane and Gressier. The area north of the national highway is characterized by alluvial plains, and the area south of the highway transitions from relatively flat agricultural land to a mountainous region. Using the water point layer from the CCMP dataset that consisted of 551 functioning water points, this study sought to confirm key elements of the initial CCMP inventory and assess ongoing operational characteristics and contamination levels of the water points in the region.

## Methods

**Figure 2.**
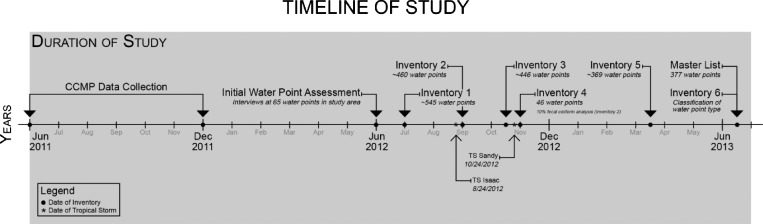
Timeline of study.

This study started with an existing dataset of 551 water points defined by the CCMP dataset using Google Earth with six inventories conducted during 2012 and 2013. Inventory 1 (Figure 2) was conducted by systematically surveying water points in the study region by moving from west to east and layering points over the original dataset of 551 until all of the original points had a corresponding point from July of 2012. Inventories 2–6 were conducted in an effort to clean the dataset and obtain additional data on the water points and water point type. Water samples were collected for inventories 2–6. For inventories 3–6, data collection used Motorola Android-based tablets and Fulcrum Spatial App (http://fulcrumapp.com), a spatial survey application. The study was reviewed and designated as exempt by the University of Florida Institutional Review Board.

For each water point, data were collected on (1) functionality of the water point (functioning/not functioning), (2) accessibility (locked/unlocked), (3) fee structure (payment/no payment), (4) organization(s) responsible for construction, and (5) entity(s) responsible for management. Global positioning system (GPS) coordinates and a digital photograph were taken at each water point, and water source type was identified and classified as improved or unimproved using the guidelines from the World Health Organization (WHO) Joint Monitoring Program (JMP).^5^

Inventory 2 occurred immediately after the passage of Tropical Storm Isaac (which became Hurricane Isaac). In inventory 2, water samples were obtained for fecal coliform analysis from a random sample of approximately 10% of water points. An attempt to collect a non-storm event baseline (including the same 10% of wells sampled for fecal coliforms in inventory 2) led to inventory 3, which occurred 6 weeks after inventory 2. Immediately after inventory 3, Tropical Storm Sandy (which became Hurricane Sandy) passed through the study area. Inventory 4 occurred immediately after the passage of Tropical Storm Sandy. This inventory only collected water samples for fecal coliform analysis from the same 10% of water points that had been sampled in inventories 2 and 3. Based on results of water sample analysis in inventories 2–4, the decision was made to obtain samples from all accessible water points in inventory 5. Inventory 6 was conducted to further classify all water points not visibly evident (from digital imagery) to be a machine-drilled borehole or India Mark II hand pump and confirm fecal coliform findings from inventory 5, focusing on well clusters with higher levels of coliform contamination.

Data were collected by four teams of local Haitian Community Health Workers (two persons per team) from the communities where the water points were inventoried. Although much of the data was collected through observational measures, in many instances, individuals at the water points contributed by providing specific information related to water point management strategies, history of functionality of the water point, and in some cases, the mobile number for the person responsible for the water point.

Community Health Worker teams collected water samples in sterilized (autoclaved) 500 mL Nalgene plastic bottles, which were returned to the University of Florida field laboratory in Gressier for analysis within 3 hours of collection. Total dissolved solids in the sample were measured by using a handheld probe (TDS-3; HM Digital, Seoul, Korea). Fecal coliform concentrations were determined by the mFC agar method.^6^ Briefly, 50-mL water samples were filtered through a 0.22-μm nylon membrane, transferred colony side up onto a petri dish with mFC agar containing rosolic acid, and incubated overnight at 44.5°C, and blue colonies growing on the plate were counted. The use of 50-mL sample volumes has a limit of detection for the presence of fecal coliforms at 2 CFU/100 mL. *Vibrio cholerae* was isolated by enrichment of a 1.5-mL water sample with 1.5 mL 2× alkaline peptone water (APW) in three tubes; tubes were incubated at 37°C and 40°C for 6–8 hours and 37°C for 18–24 hours before streak-plating on thiosulfate-citrate-bile salts (TCBS) agar as previously described.^7^ Any green or yellow colonies from the TCBS plates were subcultured onto Luria–Bertani medium agar and grown overnight at 37°C before analysis using both serology (agglutination with O1 antisera) and polymerase chain reaction (PCR) for *ompW*, *ctxA*, and *toxR*.^7^

Statistical analysis was performed with STATA MP 11. Spatial analyses were conducted to test for spatial clustering of both high and low fecal coliform concentrations relative to the mean fecal coliform concentrations over the study area. Local indicators of spatial association (LISA) using the Local Moran's *I* statistic^8^ were measured using the Spatial Statistics Toolbox in ArcGIS v10 (ESRI, Redlands, CA). The threshold distance around each water source was set at 500 m, representing a plausible community area serviced by the water source and also maximizing the absolute value of the clustering statistic (Local Moran's *I*) for all water points. This test identifies spatial clusters of water sources with either high or low contamination levels as well as spatial outliers—either clean wells surrounded by contaminated wells or vice versa. Wells were considered part of a local cluster at *P* ≤ 0.05.

## Results

The initial water point inventory conducted in July of 2012 identified 545 distinct water points, and changes from the original CCMP survey seemed to represent duplicates or new water points. Number of points counted in subsequent inventories varied because of problems with GPS localization of water points, access issues (both in terms of road conditions and whether water points were locked on subsequent inventories), and ongoing construction and other changes in water points. As of March of 2013 (inventory 5), we were able to clearly identify location and water point type for 377 water points/wells, and fecal coliform data were available for 345 water points/wells; they serve as the basis for this analysis.

Approximately 16% of wells/water points were recorded as non-functional during inventory 1. This percentage remained relatively constant through all inventories, although which water points were non-functional varied. IOs and NGOs were responsible for the construction of 56% of water points in the study region (Table 1); only 25% had evidence of a management strategy. Of those water points with evidence of a management strategy, 41 (44%) water points were managed by IO/NGOs; 19% of 213 water points constructed by IO/NGOs had evidence that the IO/NGO responsible for construction is reported to be responsible for management, whereas 27 (13%) of these water points have alternative management strategies in place, including management by schools, churches, community-based organizations, and one under the sole management of DINEPA. The remaining 145 (68%) water points lacked any sort of management strategy. We also found that 98% of water points required no payment for use and that 45% reportedly had a pump keeper responsible for the day-to-day accessibility of the water points.

There were 23 documented organizations and institutions contributing to water infrastructure in either a construction or management capacity. The rehabilitation of non-functioning water points since July of 2012 has seen a shift in organizations assuming responsibility for water points. With little programmatic continuity among IOs and NGOs in the study region, this change is likely attributed to the significantly fewer number of organizations still working on the ground in the study area on projects related to water, sanitation, and hygiene (WASH) as well as an increased presence of DINEPA. Inventory 6 revealed that many of the original IO and NGO insignias had been painted over with DINEPA's logo, and communal standpipes were upgraded from one to multiple faucets. We found the general trend for management to be for a community (*N* = 21) or an individual (*N* = 12) to assume responsibility for a water point; only 12 of 377 water points for which data were available were managed by a committee structure.^9^ Although we did find that 45% of water points had a pump keeper responsible for the water point on a daily basis, we found his/her role to be the point of contact for the water point rather than the individual responsible for management.

**Figure 3.**
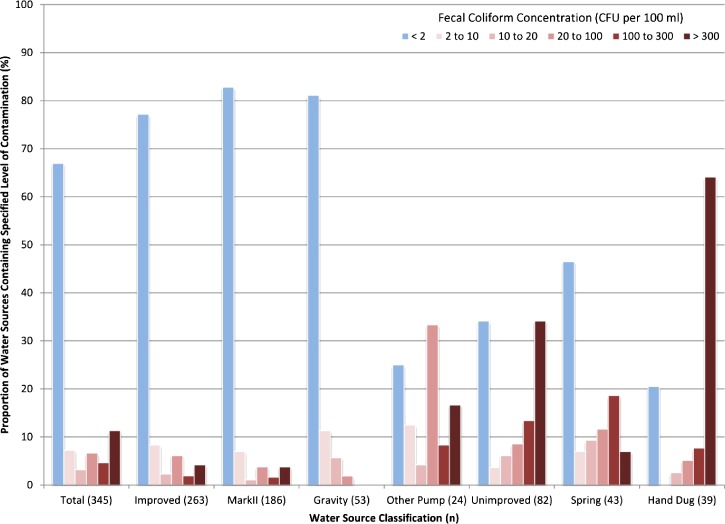
Distribution of fecal coliform concentrations from all water sources The overall distribution of water samples containing fecal coliforms at various concentrations from the limit of detection (2 CFU/100 mL) to greater than or equal to 300 CFU/100 mL is shown. The levels of fecal contamination are further divided into the improved and unimproved categories and their respective subcategories, which show the proportion of water sources in each category with the level of fecal coliform contamination specified by the legend from the least amount (dark red) to the highest levels (light pink).

**Figure 4.**
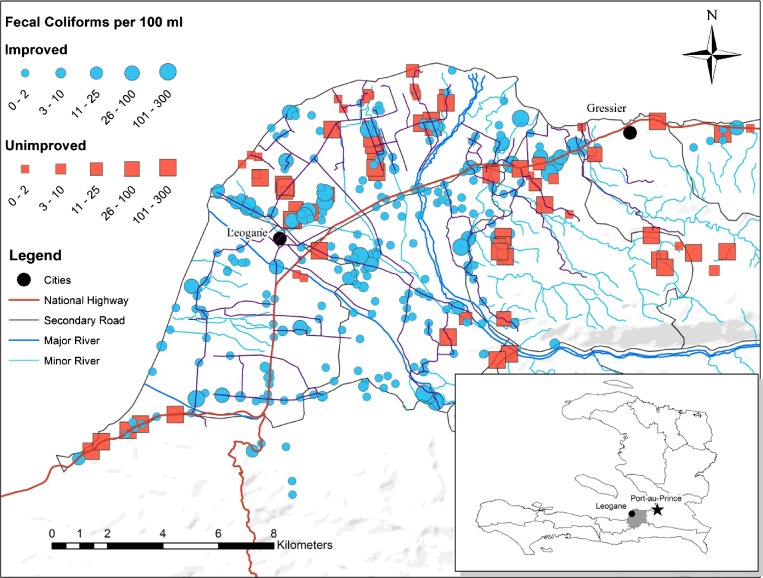
Spatial distribution of fecal coliforms by concentration (CFU per 100 mL) and source type. The geographic distribution of water sources across the study area and the overall location of the study area in Haiti (Inset) are shown. The legend shows the graduated symbols for fecal coliform concentrations (CFU per 100 mL), and the scale bar is in kilometers.

In inventory 5 (March of 2013), complete microbiologic data were obtained for 345 functioning wells in the region. Fecal coliform bacteria were identified in 37.3% of water sources. However, the proportion of water sources that contained fecal coliform bacteria was significantly higher (*P* < 0.001) among unimproved water sources compared with improved sources (80.9% versus 24.6%) (Tables 2 and 3). Comparing unimproved water sources, spring water was significantly less likely to be contaminated by fecal coliforms (53.4%) than wells dug by hand (79.4%); hand-dug wells had both the highest prevalence and the highest average concentration of fecal coliforms (208 CFU/100 mL) (Figure 3

and Table 2). Wells with India Mark II pumps were significantly less likely (*P* < 0.0001) (Table 3) to have fecal coliform bacteria (odds ratio [OR] = 0.22, 95% confidence interval [95% CI] = 0.14–0.35) and had, on average, 32 fecal coliform bacteria less than all other sources (−95% CI = −81.6 to −42.8). The spatial distribution of wells by type (improved versus unimproved) and fecal coliform counts are shown in Figure 4

**Figure 5.**
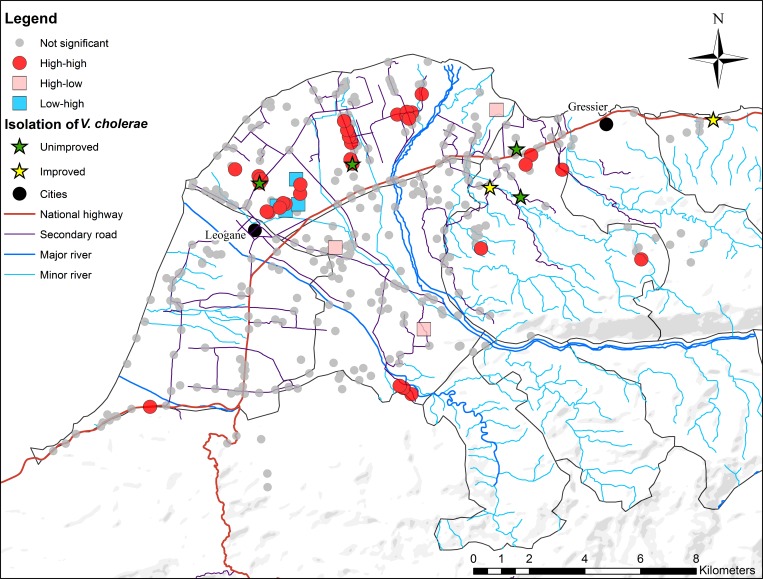
Fecal coliform spatial clusters, outliers, and the presence of *V. cholerae* non-O1. Distribution of spatial clusters and spatial outliers of water sources based on LISA analysis (red, blue, and pink dots) and the location of six *V. cholerae* non-O1 isolated from improved (yellow star) and unimproved (green stars) water sources with respect to the location of fecal coliform spatial clusters.

. Significant spatial clustering of fecal coliform-positive wells was observed using the LISA statistic, with clusters of high counts apparent in older urban areas north of Leogane city center as well as the rural areas south of Gressier and west of Leogane (Figure 5

). Spatial outliers of high fecal coliform counts were found along the coast west of Gressier and the east and southeast of Leogane. Spatial outliers of low fecal coliform counts were identified in proximity-clustered high-count wells north of the Leogane city center. Clusters with both high and low fecal coliform counts were resampled in inventory 6: results did not differ significantly from results obtained in inventory 5, providing additional evidence that these clusters identify local hotspots of high fecal coliform contamination (data not shown). There was no association between the presence or number of fecal coliforms and the presence or absence of an identifiable organization responsible for maintenance.

Toxigenic *V. cholerae* O1 (the cause of epidemic cholera) was not isolated from any of the water points sampled. However, non-toxigenic non-O1/non-O139 *V. cholerae* was isolated from six water points, all of which were fecally contaminated. Three of six water points were in immediate proximity to spatial clusters of high fecal contamination. The spatial distribution of the positive water points is shown in Figure 5.

Microbiologic data were available and analyzed for a randomly selected subset of 46 water points that were sampled serially on inventories 3–5. Inventory 3 occurred in a period between Tropical Storms Isaac and Sandy; 36% of water points had fecal coliforms. Inventory 4 occurred 36 hours after Tropical Storm Sandy, which triggered major flooding in the region: in this setting, 51% of water points were fecally contaminated, representing a significant increase over contamination rates/levels from inventory 3 (*P* = 0.03, McNemar matched pair analysis, two tail). Inventory 5 took place in March of 2013 at the end of the dry season: 34% of 46 water points were fecally contaminated at this time, which represented a significant decrease in rates/levels of coliforms from inventory 4 (*P* = 0.016, McNemar matched pair analysis, two tail) but no change from findings from inventory 3. Findings remained significant when the analysis was restricted to improved water points.

## Discussion

The average cost to have a Haitian contractor dig a well and install a pump is in the range of $7,000–$11,000 depending on depth, wellhead construction, and type of pump (E.R., personal communication). For IOs and NGOs, large numbers of wells can be dug quickly and cheaply, providing an immediate positive outcome that can be reported to funding sources. However, longitudinal maintenance costs are many times the initial cost of installation and often not accounted for in WASH budgetary allocations of IOs and NGOs.^10^,^11^ Furthermore, our inventories revealed no coordinated strategy for the water points installed after the 2010 earthquake. In the Leogane/Gressier region of Haiti immediately after the earthquake and subsequent cholera epidemic, there were at least 13 major IOs and NGOs and up to 23 organizations involved with water and water systems. This study highlights the complexity of post-earthquake water-related infrastructure development, with (1) minimal evidence of planning (including geospatial planning in terms of geography/topography/hydrology and population concentrations), (2) little to no coordination among organizations responsible for construction to assure that services were not being duplicated or missed, and (3) almost non-existent technical monitoring and management strategies (from either the public or private sectors) after wells were dug and IOs and NGOs had either departed or altered their programmatic objectives in the area. Even where it was possible to identify an organization responsible for management of a specific water point, such water points were equally likely to have fecal contamination as water points for which no management entity could be established, raising questions about the quality of management services provided and emphasizing the critical importance of actually monitoring water quality.

From a public health standpoint, the mechanically drilled boreholes with India Mark II pumps represented a substantial improvement in terms of fecal contamination compared with hand-dug wells. However, we found that improved wells, as defined by WHO, still had a 24% contamination rate with fecal coliforms during normal (non-flooding) weather conditions, and even 18% of the India Mark II pumps were fecally contaminated. There are probably multiple factors contributing to these rates of contamination. Flooding (as seen after Tropical Storm Sandy) had a significant impact on contamination, possibly because of intrusion of contaminated water into the wellhead. The lower levels of contamination that continued to be identified during the dry season (i.e., the studies conducted during inventory 5) may also result from wellhead contamination, possibly related to improper or inadequate wellhead construction. It may also reflect inadequate depth of the well. We have no data on well depth and were not able to obtain such data; to our knowledge, such data were not recorded and are not available short of an engineering assessment on a well-by-well basis.^12^ Discussions with local well contractors suggest that wells were generally in the range of 50–150 ft in depth (E.R., personal communication). If wells were of inadequate depth, there is the possibility of contamination from surface sewage outlets or possibly, contamination of shallow aquifers. We did not look specifically at other aspects of the sanitary infrastructure (such as latrine locations), which may have contributed to the risk of contamination. The identification of several local spatial clusters of fecally contaminated water points in both urban and rural areas supports these latter hypotheses. Resampling water points defined as clusters confirmed that clusters of high fecal coliforms persisted across our survey. Temporal persistence of clusters coupled with spatial outliers of highly contaminated wells surrounded by wells with low fecal coliform counts suggest that treating individual wells may reduce risk. We did not isolate toxigenic (epidemic) cholera from the wells but did see the closely related non-toxigenic, non-O1/non-O139 *V. cholerae* in association with fecal contamination, indicating that contamination of wells by epidemic strains of the microorganism is plausible.

There is inadequate medical and public health infrastructure in this region of Haiti to directly link this level of fecal contamination with occurrence of illness. Nonetheless, the finding that one-quarter of improved water points were fecally contaminated does not bode well for ongoing efforts to improve public health. Anecdotally, our Community Health Worker teams were told by people in some communities that, because of concerns about well water, surface water was used for drinking and well water was reserved for washing and bathing. However, recent studies by our group have documented the presence of toxigenic *V. cholerae* O1 at multiple surface water sampling sites,^7^ suggesting that reliance on surface water for drinking is not optimal and should be actively discouraged. Moving forward, there is an urgent need to develop a comprehensive monitoring program for water sources in the region that is ideally linked with a program of point chlorination for wells found to have fecally contaminated water as well as surface water sources. It is unclear, however, that the DINEPA has either the resources or management capabilities to undertake this massive a task.

The findings from our studies only begin to lay the foundation for determining appropriate strategies for making water sources viable over the long term.^10^,^11^ The February of 2013 National Plan for the Elimination of Cholera^13^ issued by the Haitian Ministry of Public Health and Population (MSPP) and DINEPA suggests some coordination of community-based, governmental, and private sector approaches but provides no clear plan for how this will play out in the communities affected by the complexities of water-related infrastructure. Although our findings reveal that 45% of water points had a pump keeper, giving the perception that there is a known individual responsible for the safe delivery of water in the study area, these responsible parties have no way of testing water quality and no one to whom to report non-functioning water points. However, given that pump keepers are in place, engaging communities to take responsibility for the day-to-day management of water points may be an appropriate strategy to improve well quality. Such a strategy would require proper coordination, oversight, and resource allocation by DINEPA and MSPP. Such a strategy might also be integrated with a variety of new Geographic Information System (GIS) and spatial technologies, which may make this type of monitoring and management easier in resource-limited settings.^14^

Since this inventory commenced in June of 2012, DINEPA has assumed a greater role in the region, but at the same time, most of the international investment in WASH in Gressier/Leogane has ceased. How DINEPA reconciles this complex fabric of post-earthquake water-related infrastructure into a usable network for communities that currently rely on a collection of single-point sources for access to water will determine the impacts that this infrastructure has on public health outcomes in the future. Assuring access to safe water is a challenging problem particularly confounded by the complex post-earthquake context of Haiti, where cholera remains a threat.^15^ Challenges surrounding surveillance of this water-related infrastructure cannot be addressed until a clearer understanding of what exists on the ground develops and how this infrastructure impacts the quality of water it supplies.

## Figures and Tables

**Table 1:** Summary of water point construction and management data

	Count	Percentage
Construction information		
Number constructed pre-earthquake	3	0.8
Number constructed post-earthquake	374	99.2
Total constructed	377	100.0
Constructed and marked by IO/NGO	213	56.5
Constructed with no sign of sponsor	132	35.0
Constructed by community	13	3.4
Privately constructed	16	4.2
Management strategy		
All water sources (*N* = 377)		
No evidence of management strategy	284	75.3
Evidence of management strategy	93	24.7
Managed by IO/NGO	44	47.3
Managed by community	21	22.6
Managed by elected committee	12	12.9
Managed by individuals	12	12.9
Managed by alternative	6	6.5
Managed by DINEPA	1	1.1
IO/NGO constructed water sources (*N* = 213)		
No evidence of management strategy	145	68.1
Evidence of management strategy	68	24.9
Managed by same IO/NGO	41	28.3
Managed by other Organization	27	18.6

**Table 2:** *V. cholerae* non-O1/non-O139, fecal coliforms, and total dissolved solids by well type

Parameter	*V. cholerae* non-O1 (total isolations)		Fecal coliforms (CFU/100 mL)		Total dissolved solids (ppm)	
	*n*	Count (%)	Average (SD)	Percent (positive)	*n*	Average (SD)
All types						
Improved	263	2 (0.76)	18.7 (63)	24.6	261	260 (96)
Unimproved	82	4 (4.87)	169 (134)	80.9	52	298 (152)
Improved						
Mark II	186	1 (0.53)	15.1 (59)	18.8	186	274 (99)
Gravity	53	1 (1.88)	1.6 (4.9)	18.8	52	208 (60)
Other pump	24	1 (4.16)	76.5 (108)	75	23	305 (92)
Unimproved						
Spring water	43	1 (2.35)	55.2 (91)	53.4	41	220 (78)
Dug by hand	39	2 (5.12)	208 (131)	79.4	37	327 (168)
Total	345	6 (1.74)	47.4 (100)	37.3	339	266 (108)

**Table 3:** Logistic and linear regression models showing relationships between type of water source and presence/absence and total number of fecal coliforms

Parameter	Logistic regression models					Linear regression models			
	*n*	OR	*P* value	Lower 95% CI	Higher 95% CI	Coefficient	*P* Value	Lower 95% CI	Higher 95% CI
Water sources									
Improved	263	0.08	0.000	0.04	0.15	−150.4	0.000	−172.3	−128.6
Unimproved	82	21.74	0.000	8.93	52.91	175.58	0.000	152.7	198.5
Improved									
Mark II	186	0.22	0.000	0.14	0.35	−32.23	0.000	−81.6	−42.8
Gravity	53	0.54	0.030	0.31	0.94	−47.35	0.000	−70.9	−23.8
Other pump	24	5.46	0.000	2.33	12.79	23.88	0.218	−14.2	61.9
Unimproved									
Spring water	43	2.45	0.006	1.29	4.66	11.48	0.474	−20.0	43.0
Hand-dug well	39	9.46	0.000	4.19	21.34	182.72	0.000	155.9	209.5
